# Functionally Suppressive CD8 T Regulatory Cells Are Increased in Patients with Multiple Myeloma: A Cause for Immune Impairment

**DOI:** 10.1371/journal.pone.0049446

**Published:** 2012-11-13

**Authors:** Karthick Raja Muthu Raja, Lenka Kubiczkova, Lucie Rihova, Martin Piskacek, Pavla Vsianska, Renata Hezova, Ludek Pour, Roman Hajek

**Affiliations:** 1 Babak Myeloma Group, Department of Pathological Physiology, Faculty of Medicine, Masaryk University, Brno, Czech Republic; 2 Department of Experimental Biology, Faculty of Science, Masaryk University, Brno, Czech Republic; 3 Department of Clinical Hematology, University Hospital, Brno, Czech Republic; 4 Department of Internal Medicine, Hemato-oncology, University Hospital, Brno, Czech Republic; 5 Central European Institute of Technology, Masaryk University, Brno, Czech Republic; Beth Israel Deaconess Medical Center, Harvard Medical School, United States of America

## Abstract

**Background:**

Multiple myeloma (MM) is a plasma cell malignancy frequently associated with impaired immune cell numbers and functions. In MM, several studies have previously shown that CD4 regulatory T (Treg) cells hamper effector T cell functions and enhance immune dysfunction. In this study, we aimed to prove the presence of functionally suppressive Treg cells expressing CD8 phenotype (CD8 Treg cells) in MM. To the best of our knowledge, this has not been reported previously in MM.

**Methods:**

We analyzed CD8 Treg cells and their transcription factor FoxP3 from 64 newly diagnosed MM patients using flow cytometry and real time-polymerase chain reaction (RT-PCR). RNA profile of cytokines in CD8 Treg cells was also assessed using RT-PCR. CD8 Treg cells from 5 MM patients and 5 healthy donors were functionally evaluated using proliferation assays.

**Results:**

CD8 Treg cells (CD8+CD25hi+) were significantly elevated in MM patients (P<0.0001), and their transcription factor FoxP3 expression was also higher in MM (P<0.0001) compared to healthy donors which was evidenced by flow cytometry and RT-PCR analyses. CD8 Treg cells negatively correlated with total lymphocyte count (P = 0.016). Functional studies revealed that CD8 Treg cells isolated from MM patients and healthy donors inhibited proliferation of CD4 T cells in a concentration dependent manner. In the presence of CD8 Treg cells in proliferation assays, level of IFN-γ was decreased but not IL-10. CD4 T cells from MM patients secreted abnormal level of IL-10 compared to healthy donors (P = 0.01) in proliferation assays without CD8 Treg cells. RNA profile of cytokines from CD8 Treg cells did not differ significantly between MM patients and healthy donors.

**Conclusions:**

These findings show the presence of increased number of functionally suppressive CD8 Treg cells in MM patients. We believe that these suppressive CD8 Treg cells might enhance immune impairment and disease progression in MM.

## Introduction

Multiple myeloma (MM) is a malignancy of plasma cells (PCs); it is the second most common hematological malignancy next to lymphoma. MM is typically characterized by infiltration of ≥10% of PCs, ≥30 g/L of monoclonal protein and presence of CRAB symptoms (hypercalcemia, renal insufficiency, anemia and bone lesions). Immune dysfunction is also an important feature of MM, which promotes tumor progression, infections and resistance to chemotherapy [1]. Several studies in MM confirmed that CD4 T regulatory (Treg) cells were elevated and functional in suppressing the effector T cells [2], [3], [4]. Elevated numbers of CD4 Treg cells have been found to enhance tumor progression and poor survival in various hematological (including MM) and non-hematological malignancies [5], [6], [7], [8]. Very recently, research effort has been aimed at screening the presence of CD8 Treg cells in pathological conditions, including cancer. Two major categories of CD8 Treg cells have been described. The first one is non-specific immune suppression (mediated by CD8+CD25+, CD8+CD122+, CD8+CD45RClow), the second is antigen specific immune suppression (mediated by CD8+CD28−, CD8+CD75s+, CD8+CD45RChi TC1 and TCR peptide specific CD8αα Treg cells) [9]. These CD8 Treg cells mediate suppression through various mechanisms including release of IL-10 cytokine (CD8+CD122+ Treg), interfering with antigen presenting cells (CD8+CD28− Treg) and contact dependent mechanism via cytotoxic T lymphocyte antigen-4 (CTLA-4) and transforming growth factor-β (TGF-β) (CD8+CD25hi+FoxP3+ Treg) [10], [11], [12]. Similarly to CD4 Treg cells, CD8 Treg cells also express FoxP3, TNFRII, CCR8, TGF-β and CTLA-4 markers [11]. Previously, few studies reported the presence of CD8 Treg cells in prostate, nasopharyngeal and colorectal cancers and these cells actively suppressed T cell proliferation [13], [14], [15]. In this study, we extensively studied the number, phenotype, functional activity, FoxP3 gene expression and cytokine profile of CD8 Treg cells in newly diagnosed MM patients. More importantly, we aimed to prove the presence of a new regulatory subset of cells with CD8 phenotype in MM patients and their suppression on CD4 T cells.

## Methods

### Patients and Healthy Subjects

A cohort of 64 previously untreated or newly diagnosed MM patients were included in this study after obtaining the signed informed consent form according to the Helsinki protocol. The study was approved by University Hospital Brno institutional review board. Both peripheral blood (PB) and bone marrow (BM) samples were collected and used in this study. The median age of the patient cohort was 63 years (range: 39–83). Patients’ baseline clinical characteristics are summarized in Table 1. Eighteen age-matched healthy donors PB samples were also collected and used as controls. The median age of the healthy donor cohort was 64 years (range: 54–72).

**Table 1:** Patients’ baseline characteristics.

	n = 64
Age, years; median (range)	63 (39–83)
Male/Female; % (n)	55% (35)/45% (29)
**International staging system (ISS); % (n)**	
ISS-1	52% (33)
ISS-2	20% (13)
ISS-3	23% (15)
Unavailable	5% (3)
**Myeloma subtype; % (n)**	
IgG	66% (42)
IgA	14% (9)
Light chain	8% (5)
Others (non-secretory, oligoclonal)	9% (6)
Unavailable	3% (2)
β2-microglobulin mg/L; median (range)	3.00 (1.45–16.30)
Albumin g/dL; median (range)	4.03 (20.90–51.50)
Creatinine mg/dL; median (range)	1.00 (0.62–8.40)
Calcium mg/dL; median (range)	9.12 (7.40–11.48)
Hemoglobin g/dL; median (range)	11.6 (6.01–16.30)
Serum monoclonal protein g/L; median (range)	25.4 (0–85.2)
% of bone marrow plasma cells; median (range)	2.20 (0.02–49.70)

### Antibodies and Fluorescence Activated Cell Sorter (FACS) Analysis

For staining, the following fluorescent conjugated monoclonal antibodies were used: CD8-APC, CD25-PE-Cy7, (CTLA-4, CD62L, CD45RO, CD127, CD28)-PE and FoxP3-FITC (antibodies obtained from BD Biosciences, Beckman Coulter, EXBIO and ebioscience). Surface staining was performed for CD8, CD25, CD28, CD45RO, CD62L, CTLA-4 and CD127 antigens. For detection of FoxP3 molecule, intracellular staining was done according to eBioscience protocol (eBioscience). Labeled cells were measured and data were acquired on FACS Canto II (BD Biosciences). Acquired data were analyzed using FACSDIVA 6.1.2 software (BD Biosciences).

### Isolation of CD8 T Regulatory Cells and CD4 T cells

Total T lymphocyte population was isolated from peripheral blood mononuclear cells (PBMCs) using Pan T Cell Isolation Kit II (Miltenyi Biotech). Isolated T lymphocytes were labeled with CD4-PerCp-Cy5.5, CD8-APC and CD25-PE-Cy7 and sorted into two fractions: CD8+CD25hi+ cells and CD4+CD25− cells using FACS Aria (BD Biosciences).

### Assessment of Inhibitory Function of CD8 Treg Cells

To assess the inhibitory activity of CD8 Treg cells against CD4 T cells (CD4+CD25−), CFSE (carboxyfluorescein succinimidyl ester) based proliferation assay was performed. Isolated CD4 T cells were stained with CFSE. Labeled cells were plated into 24-well round bottom plates at a concentration of 10^5^ cells/well. CD4 T cells were stimulated with anti-CD3 and anti-CD28 beads at a ratio of 1∶1 (cells: beads). Then, isolated CD8 Treg cells were co-cultured in different proportions with stimulated CD4 T cells (1∶1, 1∶5, 1∶10, 0∶1; CD8 Treg cells: CD4 T cells) in a final volume of 200 µL of T cell expansion basal medium containing 10% of fetal calf serum, 2 mM L-glutamine and 100 U/ml penicillin-streptomycin (all obtained from Gibco Invitrogen). Proliferation of CD4 T cells was measured by dilution of CFSE on flow cytometer after 4 days of culturing at 37°C in 5% of CO_2_ atmosphere.

### Profiling of Cytokines from Proliferation Assays

Supernatants from proliferation assays were collected and analyzed for IFN-γ and IL-10 cytokines using human cytometric bead array flex sets (BD Biosciences). Samples were prepared according to manufacturers’ recommendation and measured on FACS Array (BD Biosciences). Data were analyzed using FCAP Array software (BD Biosciences).

### Evaluation of FoxP3 Expression Using RT-PCR

Total RNA was extracted from isolated CD4 Treg cells, CD8 Treg cells and non-regulatory T cells (CD3+CD25−) by RNeasy Mini Kit (Qiagen). cDNA was synthesized from 100 ng total RNA using High Capacity Reverse Transcription Kit (Applied Biosystems) according to manufacturers’ recommendation in a final volume of 20 µl. Real time polymerase chain reaction (RT-PCR) was performed on StepOne Real-Time PCR System (Applied Biosystems) using FoxP3 gene specific assay (Applied Biosystems Hs01085834_m1). Data were normalized to human glyceraldehyde phosphate dehydrogenase (GAPDH, Applied Biosystems 4310884E) for each sample. Briefly, reactions were performed in 48 well plates with 12.5 µl of TaqMan Gene Expression Master Mix, 7.5 µl of sterile water, 1.25 µl of primer/probe reagent for FoxP3, 1.25 µl of primer/probe reagent for GAPDH and 2.5 µl of cDNA template at a total reaction volume of 25 µl. Cycles included one 2-min hold (50°C), a 10-minute denaturation (95°C), 40 cycles of denaturation (95°C for 15 seconds) and annealing/extension (60°C for 1 min). Raw data were analyzed and expression of FoxP3 was calculated using 2^−dCq^ formula. On 2% agarose gel, PCR products were analyzed by electrophoresis (as a marker, 100 bp DNA ladder from Fermentas, SM0243 was used).

### Evaluation of Cytokines from CD8 T Regulatory Cells Using RT-PCR

Isolated CD8 Treg cells (CD8+CD25hi+) and non-regulatory CD8 T cells (CD8+CD25−) were stimulated with phorbol 12-myristate 13-acetate (PMA) plus ionomycin (ebioscience) for 24 hours. After stimulation, total RNA was extracted using RNeasy Mini Kit (Qiagen). cDNA synthesis and RT-PCR with specific probes for IL-2, IL-4, IL-6, IL-10, IL-13, IFN-γ, TNF-α, TGF-β and GAPDH (Applied Biosystems: Hs00174114_m1, Hs00174122_m1, Hs00174131_m1, Hs00961622_m1, Hs00174379_m1, Hs99999041_m1, Hs00174128_m1, Hs00998133_m1) were performed and analyzed as described above.

### Immunoblotting

Sorted CD4 Treg cells, CD8 Treg cells and non-regulatory T cells (CD3+CD25−) were mixed with 2× Laemmli sample buffer, and immediately boiled for 2 min and stored at −20°C. Proteins from denatured cells for each sample were separated by polyacrylamide gel electrophoresis and transferred into nitrocellulose membrane. Then, the membrane was blocked in Tris buffer saline (pH 7.4) with 0.1% Tween 20 (TBST) containing 5% of non-fat milk. Separated proteins were probed with primary antibodies including, mouse anti-human FoxP3 (1∶1000, clone hFOXY, eBioscience) and rabbit anti-human GAPDH (1∶2000, clone 14C10, Cell Signaling Technology) overnight at 4°C in TBST buffer. Subsequently, membrane was washed in TBST and incubated with secondary antibodies conjugated with horseradish peroxidase (anti-mouse IgG and anti-rabbit IgG, 1∶10000, Sigma-Aldrich) in TBST buffer and visualized by enhanced chemiluminescence (SuperSignal, Thermo scientific).

### Statistical Analysis

Mann-Whitney U test and Student t test were used to estimate the statistical significant difference between independent groups. Spearman correlation was used to compare the association between variables. P value of <0.05 was considered as statistically significant.

## Results

### Phenotypic Characterization of CD8 T Regulatory Cells

**Figure 1:**
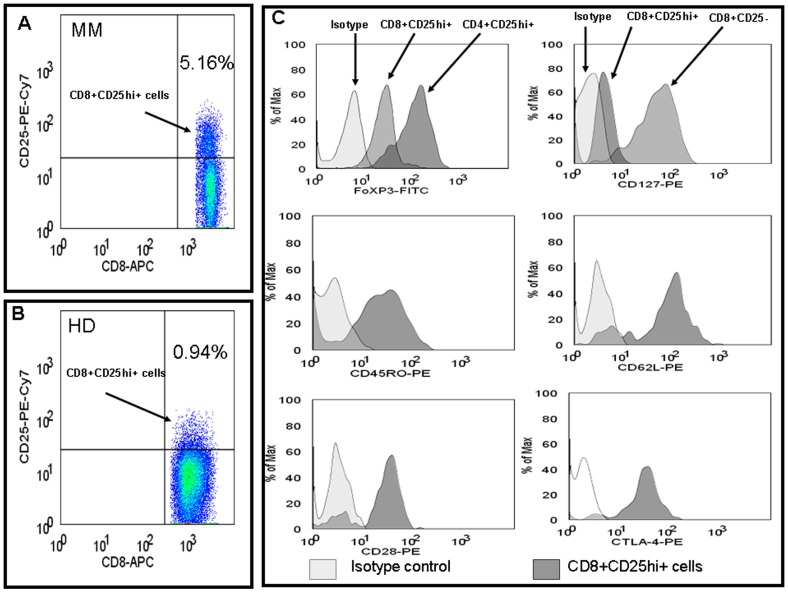
Phenotypic feature of CD8 T regulatory cells. Flow cytometric analyses of CD8 Treg cells (CD8+CD25hi+) from a MM patient (A) and a healthy donor (B) are presented. (C) Represents phenotypic profile of CD8 Treg cells (CD8+CD25hi+) from a MM patient. At first, CD8 Treg cells were identified and further analyzed for intracellular FoxP3 and various surface antigens, including CD127, CD62L, CTLA-4, CD45RO and CD28. Expression of these antigens is shown on respective histograms. CD4+CD25hi+ Treg cells served as positive control for expression of FoxP3. Positive expression of specific marker/antigen is indicated as dark grey peak, and isotype control is indicated as light grey peak. MM, multiple myeloma; HD, healthy donor; FoxP3, forkhead-winged helix transcription factor; CTLA-4, cytotoxic T lymphocyte antigen-4.

CD8 T cells expressing high intensity of CD25 molecule were considered as CD8 Treg cells (CD8+CD25hi+). These CD8 Treg cells expressed FoxP3, CTLA-4, CD45RO, CD62L and CD28 but not CD127. Positive expression of these antigens ranged from dim to high intensity (Fig. 1).

### CD8 T Regulatory Cell Numbers are Increased in Multiple Myeloma

Frequency and absolute number of PB CD8 Treg cells from MM patients were increased compared to healthy donors (Table 2). We also analyzed frequency and absolute number of total lymphocyte population and CD8 T cells; the results showed that total lymphocytes but not CD8 T cells were significantly reduced in MM patients compared to healthy donors (Table 2). To enumerate FoxP3 expression in CD8 Treg cells, we quantified it from CD8 T cells co-expressing CD25hi and FoxP3. Similarly to CD8 Treg cells, frequency and absolute number of CD8 T cells co-expressing CD25hi and FoxP3 were increased in MM patients compared to healthy donors (Table 2). Further, we found that CTLA-4 expression in CD8 Treg cells from MM patients (n = 12) was higher compared to healthy donors (n = 5) (median, 69.3% vs. 44.6%; P = 0.039).

**Table 2:** Frequency and absolute number of peripheral blood CD8 T regulatory cells, CD8.

Cell types	Frequency in %, median(range)			Absolute number/µl, median(range )		
	MM	HD	Pvalue	MM	HD	Pvalue
CD8 Treg cells	5.80(0.29–22.57)	0.40(0.14–1.16)	<0.0001	24(2–179)	2(0.5–7)	<0.0001
CD8 T cellco-expressing CD25hiand FoxP3	0.42(0.03–1.67)	0.15(0.07–0.35)	<0.0001	1.46(0.16–10.96)	0.74(0.25–2.58)	<0.0001
CD8 T cells	26.74(1.50–67.76)	18.37(9.29–29.46)	0.009	495(55–2328)	451(268–777)	0.20
Total lymphocytes	15.7(2.97–73.62)	38.81(21.55–51.60)	<0.0001	1840(800–3840)	2815(1920–3690)	<0.001

T cells and total lymphocytes from multiple myeloma patients and healthy donors.

Statistical difference was tested by Mann-Whitney U test.

In BM samples, median frequency and absolute number of CD8 Treg cells and CD8 T cells co-expressing CD25 and FoxP3 were observed as 5.35%, 25 cells/µL and 0.40%, 1.9 cells/µL, respectively. Unfortunately, we did not have healthy BM samples to compare with MM patients.

### Increased CD8 T Regulatory Cell Numbers are Associated with Reduced Total Lymphocytes

Correlation analysis showed that the number of CD8 Treg cells and CD8 T cells co-expressing CD25hi and FoxP3 from PB and BM of MM patients was significantly correlated (r = 0.70; P<0.0001, r = 0.51; P<0.0001, respectively). We observed a negative association between number of CD8 Treg cells and total lymphocytes from both PB and BM (r = −0.31; P = 0.016, r = −0.33; P = 0.007, respectively). Statistically, insignificant negative association was observed between number of CD8 Treg cells and CD8 T cells from both PB and BM (data not shown). Next, we analyzed correlation between CD8 Treg cells and CD4/CD8 ratio. Data of CD4/CD8 ratio was available from 12 MM patients (median CD4/CD8 ratio: 2.55); correlation analysis showed insignificant negative correlation between CD8 Treg cells and CD4/CD8 ratio (data not shown). These data suggest that expansion of CD8 Treg cells enhance immune suppression which is evidenced by reduced total lymphocytes.

We did not find any significant association between CD8 Treg cells and clinical stages or clinical features of MM (data not shown). Also, neither age nor gender dependent increase/association was observed for CD8 Treg cells in MM patients and healthy donors (data not shown).

### CD8 T Regulatory Cells are Potent Suppressors of CD4 T Cells

**Figure 2:**
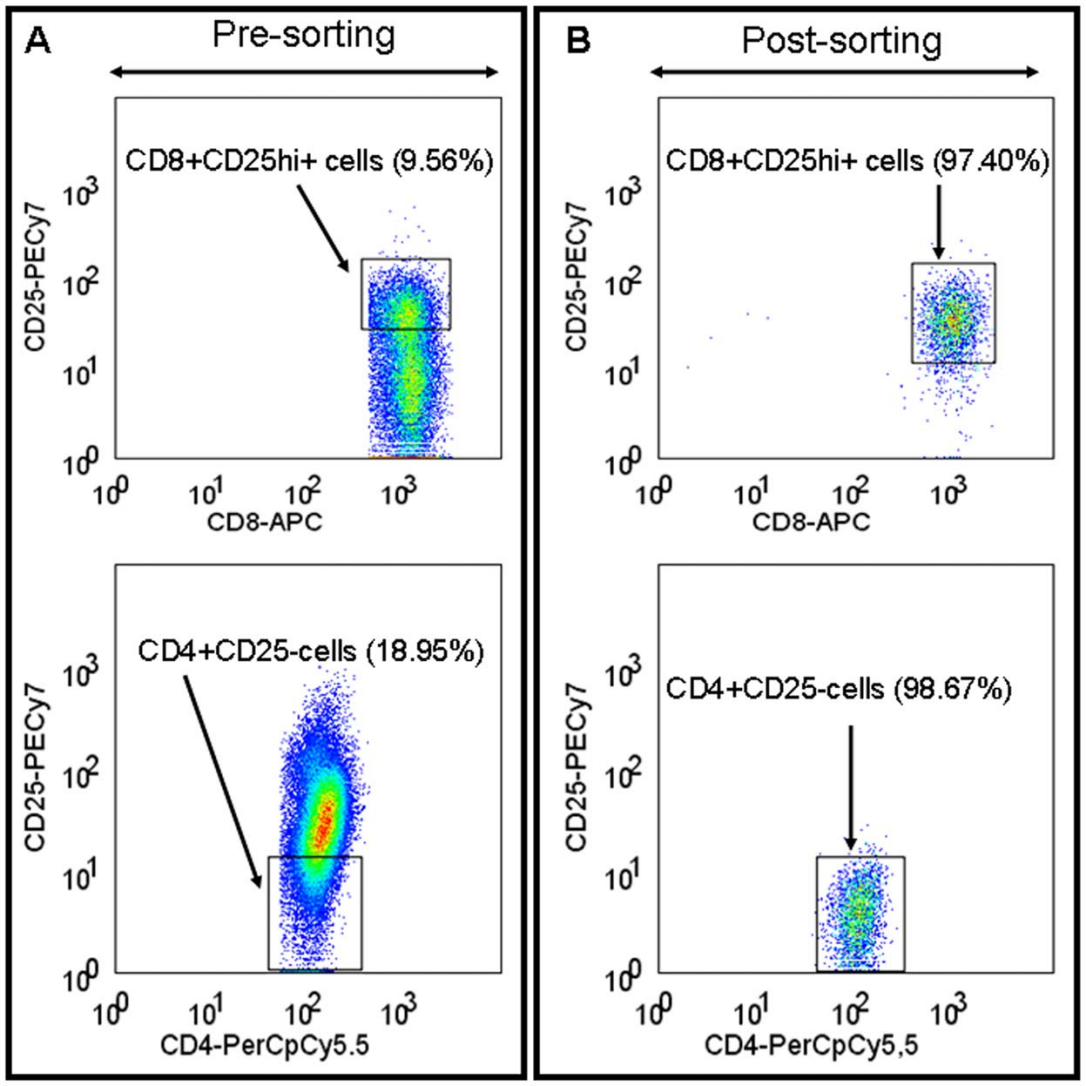
Isolation of CD8+CD25hi+ (CD8 Treg cells) and CD4+CD25− cells. Isolated T lymphocytes were labeled with fluorescent conjugated monoclonal antibodies and sorted by FACS Aria into CD8+CD25hi+ cells and CD4+CD25− cells. As an example, pre (A) and post (B) sorted CD8+CD25hi+ cells and CD4+CD25− cells are shown from a MM patient, and their purity is expressed in percentage.

**Figure 3:**
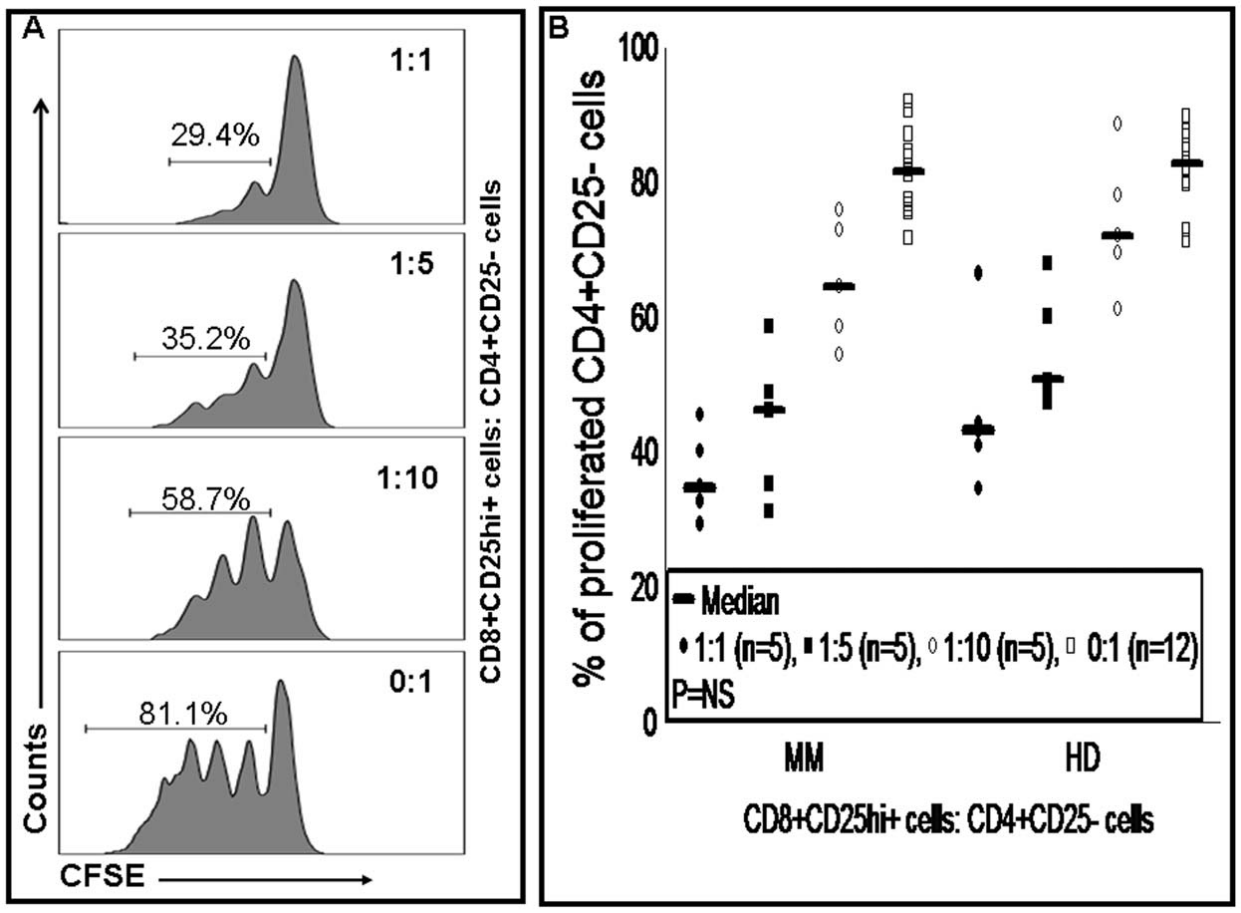
Functional analysis of CD8 T regulatory cells. CD4 T cells were labeled with CFSE and co-cultured with CD8 Treg cells in different ratios (1∶1, 1∶5, 1∶10 and 0∶1; CD8 Treg cells to CD4 T cells) to demonstrate the suppressive function of CD8 Treg cells. After 4 days of co-culturing, proliferation of CD4 T cells was examined by flow cytometer for dilution of CFSE in the FITC channel. (A) Represents proliferation assay from a MM patient. Proliferation of CD4 T cells was inhibited by CD8 Treg cells in a concentration dependent manner but not in their absence, which was clearly shown by the dilution of CFSE. Level of CD4 T cell proliferation in the presence or absence of CD8 Treg cells is represented in percentage. (B) Comparison of MM patients and healthy donors for inhibition of CD4 T cell proliferation mediated by CD8 Treg cells at different concentrations. Mann-Whitney U test showed no significant difference in proliferation assays between MM patients and healthy donors either in the presence or absence of CD8 Treg cells. Median proliferation of CD4 T cells is indicated by horizontal line, small dots and squares represent raw data from each experiment. MM, multiple myeloma; HD, healthy donor; NS, statistically not significant.

Proliferation assays were performed using highly purified cells (CD8+CD25hi+ and CD4+CD25−) with a purity of ≥96% for all samples (Fig. 2). We evaluated the inhibitory activity of CD8 Treg cells (CD8+CD25hi+) from 5 MM patients and 5 healthy donors against CD4 T cells in CFSE based proliferation assays (Fig. 3A). We found that CD8 Treg cells isolated from MM patients and healthy donors inhibited CD4 T cell proliferation in a concentration dependent manner. In comparative analysis, CD8 Treg cell mediated proliferation inhibition of CD4 T cells did not differ significantly between MM patients and healthy donors (P>0.05) (Fig. 3B). Next, we intended to analyze the proliferation potential of CD4 T cells from MM patients (n = 12) and healthy donors (n = 12) in proliferation assays without CD8 Treg cells. Analysis clearly showed that proliferation potential of CD4 T cells was comparable between MM patients and healthy donors (Fig. 3B).

### CD8 T Regulatory Cells Suppress IFN-γ Secretion

**Figure 4:**
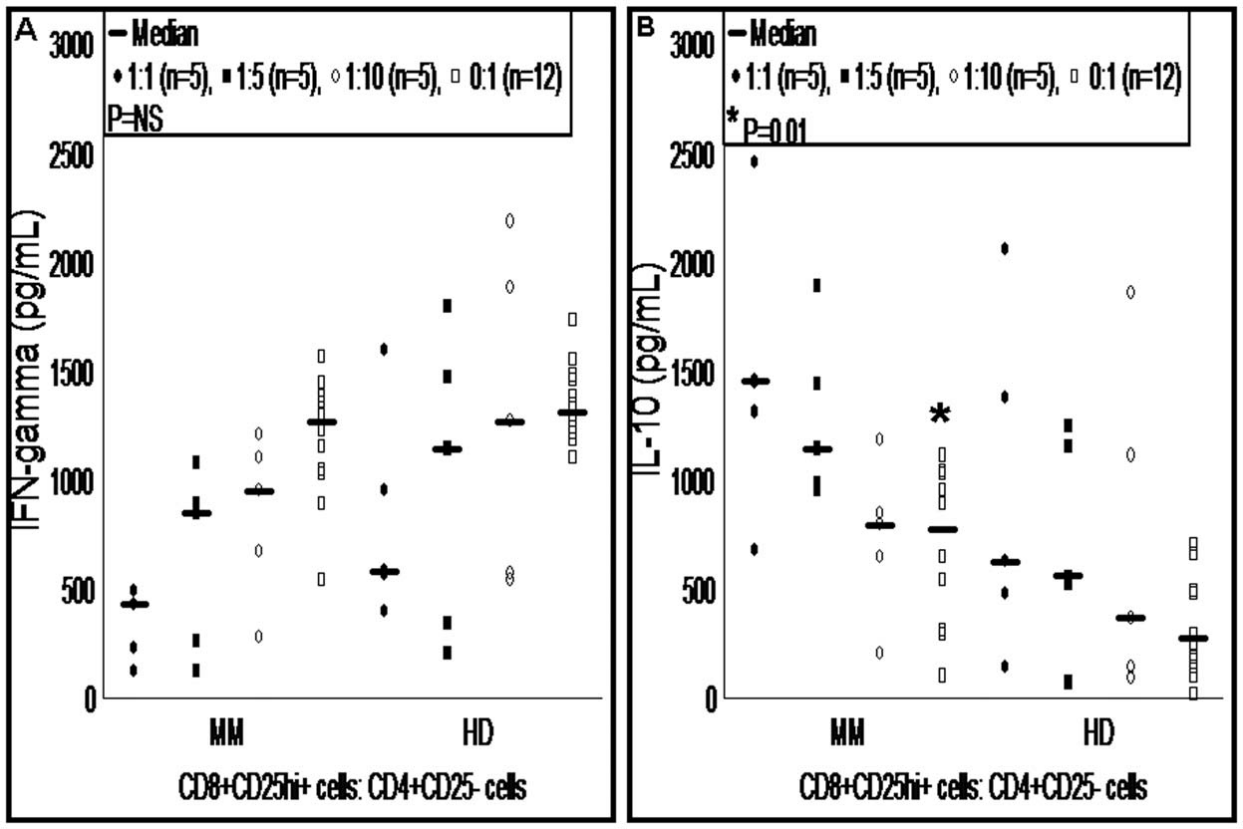
Profiling of pro- and anti-inflammatory cytokines. Culture supernatants from proliferation assays were collected after 4 days of culturing and profiled for IFN-γ and IL-10 cytokines using cytometric bead array assays. Level of IFN-γ (A) and IL-10 (B) cytokines are shown from MM patients and healthy donors. Mann-Whitney U test showed significant difference only in the level IL-10 between MM patients and healthy donors (*P = 0.01) from proliferation assays without CD8 Treg cells. Median value of cytokines is indicated by horizontal line, small dots and squares represent raw data from each experiment. MM, multiple myeloma; HD, healthy donor; IFN-γ, interferon-gamma; IL-10, interleukin-10; *, statistically significant.

To further validate the immunosuppressive ability of CD8 Treg cells, we assessed pro-inflammatory (IFN-γ) and immunosuppressive (IL-10) cytokines from supernatant of proliferation assays. Consistent with proliferation inhibition, IFN-γ levels were reduced in MM patients and healthy donors according to the number of CD8 Treg cells added into proliferation assays. However, in the absence of CD8 Treg cells, IFN-γ levels were increased (Fig. 4A). IFN-γ secretion from proliferation assays of MM patients and healthy donors did not differ significantly in the presence or absence of CD8 Treg cells. IL-10 levels were increased depending on the number of CD8 Treg cells added into proliferation assays. The levels of IL-10 did not differ significantly between MM patients and healthy donors (Fig. 4B). However, proliferation assays without CD8 Treg cells from MM patients showed a 3-fold increase in the level of IL-10 compared to healthy donors (median pg/mL, 775.85 vs. 274.90; P = 0.01). This abnormal secretion of IL-10 cytokine from MM patients might be due to CD4 T cell impairment.

### FoxP3 Expression is Increased in CD8 T Regulatory Cells of Multiple Myeloma Patients

**Figure 5:**
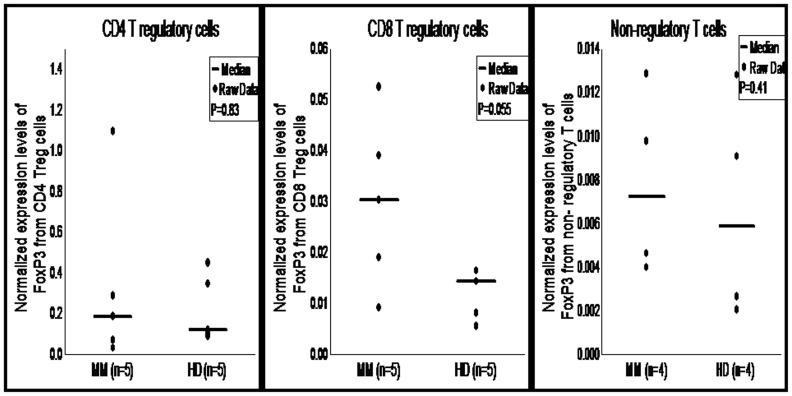
Expression profile of FoxP3 in CD4 and CD8 T regulatory cells. Total RNA isolated from purified CD4 Treg cells, CD8 Treg cells and non-regulatory T cells was analyzed for FoxP3 expression using RT-PCR. Mann-Whitney U test showed closer statistical significant difference in the expression of FoxP3 in CD8 Treg cells between MM patients and healthy donors (P = 0.055) but not in CD4 Treg cells and non-regulatory T cells. Median value of cytokines is indicated by horizontal line, small dots and squares represent raw data from each experiment. MM, multiple myeloma; HD, healthy donor; FoxP3, forkhead-winged helix transcription factor, Treg cells; T regulatory cells.

**Figure 6:**
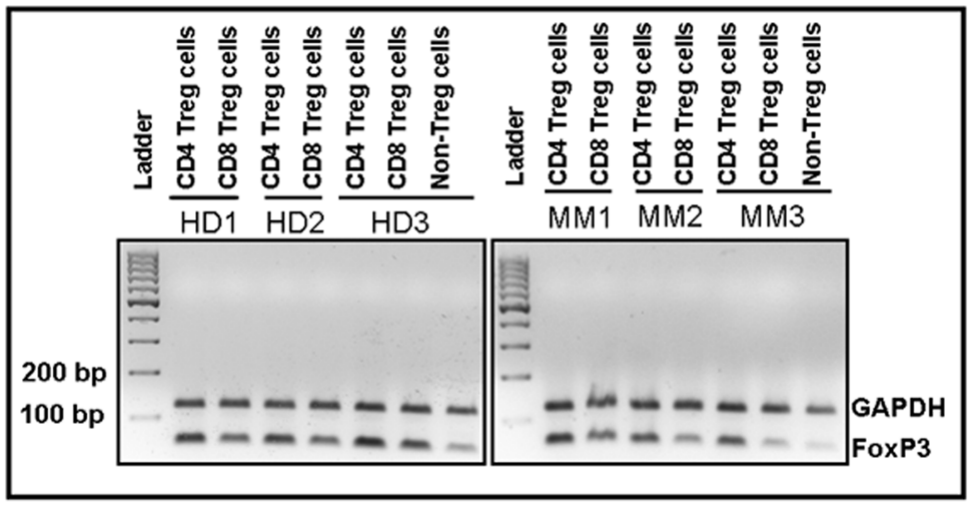
Analysis of PCR products using agarose gel electrophoresis. After RT-PCR, FoxP3 and GAPDH PCR products were analyzed using agarose gel (2%) electrophoresis. Three HD and three MM patients PCR products from CD4 Treg cells and CD8 Treg cells are shown. Also, FoxP3 and GAPDH PCR products from non-regulatory T cells of a MM patient and a healthy donor are presented. PCR products, 61 bp and 118 bp represent FoxP3 and GAPDH, respectively. Treg cells, T regulatory cells; Non-Treg cells; non-regulatory T cells; MM, multiple myeloma; HD, healthy donor; FoxP3, forkhead-winged helix transcription factor; GAPDH, human glyceraldehyde 3-phosphate dehydrogenase.

FoxP3 gene encodes a forkhead-winged helix transcription factor. This factor plays a significant role in development, function and identification of Treg cells. Using RT-PCR, we analyzed FoxP3 expression in CD4 Treg cells and CD8 Treg cells isolated from 5 MM patients and 5 healthy donors (Fig. 5). Further, PCR products were confirmed on agarose gel (Fig. 6). RT-PCR data showed higher FoxP3 expression in CD8 Treg cells isolated from MM patients compared to healthy donors (P = 0.055). However, FoxP3 expression in CD4 Treg cells from MM patients and healthy donors did not differ significantly (P = 0.83) (Fig. 5). In comparative analysis, FoxP3 expression in CD4 Treg cells was significantly increased compared to CD8 Treg cells in both MM patients (median: 0.184 vs. 0.030; P = 0.036) and healthy donors (median: 0.119 vs. 0.014; P = 0.012). When we compared FoxP3 expression in CD4 Treg cells and CD8 Treg cells with non-regulatory T cells (median FoxP3 expression in non-regulatory T cells: MM-0.0072, HD-0.0058) from MM patients and healthy donors; results showed that CD4 Treg cells had significantly increased expression of FoxP3 compared to non-regulatory T cells (MM: P = 0.019, HD: P = 0.019). But CD8 Treg cells had insignificant increase in the expression of FoxP3 compared to non-regulatory T cells (MM: P = 0.06, HD: P = 0.19).

Of note: Ct values for GAPDH and FoxP3 are summarized in supplementary table (Table S1).

### RNA Cytokine Profile of CD8 T Regulatory Cells

We analyzed the RNA profile of cytokines (1L-2, IL-4, IL-6, IL-10, IL-13, IFN-γ, TGF-β and TNF-α) in CD8 Treg cells stimulated with PMA plus ionomycin from MM patients (n = 3) and healthy donors (n = 3) by RT-PCR. RNA profile of cytokines showed no significant changes between MM patients and healthy donors but a trend of increase was observed for several cytokines (IL-2, IFN-γ, TGF-β and TNF-α) in CD8 Treg cells from healthy donors compared to MM patients (Table 3). Expression of IL-10 and IL-6 cytokines was insignificantly increased in MM patients than in healthy donors. Next, we compared RNA profile of cytokines in non-regulatory CD8 T cells (CD8+CD25−) stimulated with PMA plus ionomycin from MM patients and healthy donors. Comparative analysis showed no significant changes between MM patients and healthy donors in non-regulatory CD8 T cells (Table 3). Expression of IL-4 cytokine was undetectable in both CD8 Treg and non-regulatory CD8 T cells from MM patients and healthy donors.

**Table 3:** RNA profile of cytokines in CD8 T regulatory and non-regulatory CD8 T cells from multiple myeloma patients and healthy donors.

CD8 Treg cells	MM (n = 3),mean (±SD)	HD (n = 3),mean (±SD)	P value	non-regulatory CD8 T cells	MM (n = 3),mean (±SD)	HD (n = 3),mean (±SD)	P value
IL-2	0.0404 (0.0145)	0.7848 (0.6010)	0.10	IL-2	0.3075 (0.1879)	0.2704 (0.2021)	0.66
IL-6	0.0098 (0.0162)	0.0026 (0.0016)	0.54	IL-6	0.0015 (0.0015)	0.0019 (0.0015)	0.10
IL-10	0.0014 (0.0015)	0.0005 (0.0003)	0.66	IL-10	0.0004 (0.0007)	0.0002 (0.0001)	0.46
IL-13	0.0119 (0.0042)	0.0178 (0.0104)	0.66	IL-13	0.0015 (0.0011)	0.0008 (0.0007)	0.38
IFN-γ	0.0661 (0.0769)	0.6927 (0.6150)	0.38	IFN-γ	0.4126 (0.6286)	0.5042 (0.3335)	0.66
TGF-β	0.1260 (0.0323)	0.3499 (0.1837)	0.10	TGF-β	0.3046 (0.2368)	0.4968 (0.4397)	0.10
TNF-α	0.0710 (0.0199)	0.5216 (0.2559)	0.10	TNF-α	0.2124 (0.3010)	0.4014 (0.0750)	0.40

Raw data were analyzed and calculated using 2^−dCq^ formula. Statistical difference was tested by Student t-test. SD, standard deviation.

### FoxP3 Protein in CD8 T Regulatory Cells Evaluated by Immunoblotting

**Figure 7:**
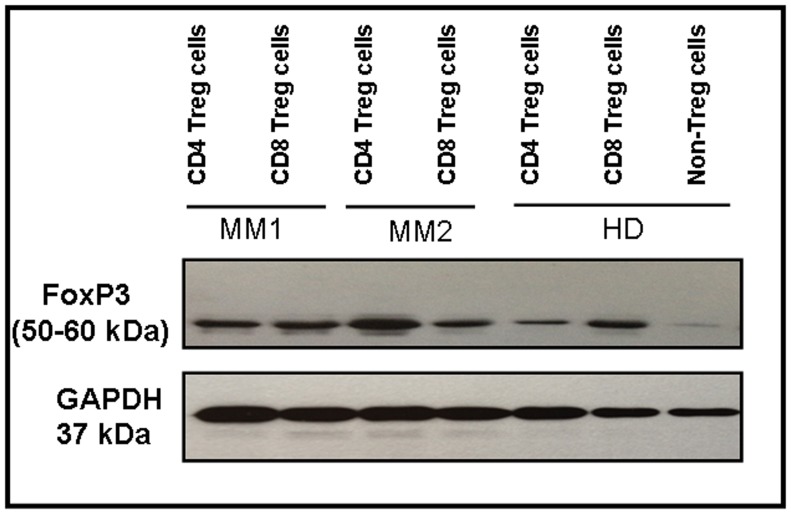
Evaluation of FoxP3 protein in CD4 and CD8 T regulatory cells using immunoblotting. CD4 Treg cells and CD8 Treg cells were denatured and proteins were separated by polyacrylamide gel electrophoresis. FoxP3 and GAPDH proteins were detected by immunoblotting. FoxP3 (top row) and GAPDH (bottom row) protein levels in CD4 Treg cells and CD8 Treg cells from 2 MM patients and a healthy donor are shown. As a control, FoxP3 and GAPDH protein levels in non-regulatory T cells are shown from a healthy donor. Treg cells, T regulatory cells; Non-Treg cells; non-regulatory T cells; MM, multiple myeloma; HD, healthy donor; FoxP3, forkhead-winged helix transcription factor; GAPDH, human glyceraldehyde 3-phosphate dehydrogenase.

To confirm the flow cytometry findings of FoxP3 expression in CD8 Treg cells, we analyzed the level of FoxP3 protein from CD4 Treg cells and CD8 Treg cells using immunoblotting. Results showed that both CD4 Treg cells and CD8 Treg cells from MM patients and healthy donors had higher level of FoxP3 protein (50–60 kDa) (Fig. 7). However, non-regulatory T cells (CD3+CD25−) showed lower level of FoxP3 protein.

## Discussion

There are several mechanisms behind tumor driven immune evasion. One of the mechanisms is Treg cells dependent immune suppression or evasion which had been shown and accepted both in hematological and non-hematological malignancies [16]. Studies have shown that several tumor-derived molecules are (CCL17, CC22, prostaglandin E2) involved in recruitment of CD4 Treg cells at tumor bed or periphery to induce tolerance against anti-tumor responses [17], [18].

We are the first to demonstrate that functionally active CD8 Treg cells are elevated in MM patients, which has been previously reported only in solid tumors including prostate, colorectal and nasopharyngeal cancers [13], [14], [15]. In our study, we have shown that in PB of newly diagnosed MM patients, CD8 Treg cells were elevated. Also, the number of FoxP3 expressing CD8 Treg cells was increased. CD8 Treg cells expressed various phenotypic markers including CD45RO (memory marker), CD62L, CTLA-4, CD28 and CD127^dim/negative^. We did not observe strong expression of all these antigens, but their expression ranged from dim to positive. This observation was consistent with Chaput et al finding [15]. Also, CD62L and CTLA-4 expression was previously shown in CD4 Treg cells from MM patients [2]. Expression of CD45RO, CD62L and CTLA-4 molecules are associated with activation and suppressive function of Treg cells [11], [19], [20]. Current study also showed increased CTLA-4 expression in CD8 Treg cells from MM patients. This suggests potent suppressive feature of CD8 Treg cells. There is also another subset of CD8 Treg cells which do not express CD28 and suppress T cells in IL-10 dependent manner but two different studies claim opposing results with regard to FoxP3 gene expression [21], [22].

Evaluation of FoxP3 expression by RT-PCR showed higher expression of FoxP3 in CD8 Treg cells of MM patients compared to healthy donors, which is also confirmed by flow cytometry finding. Interestingly, we did not find similar expression between CD4 Treg cells and CD8 Treg cells. CD4 Treg cells expressed increased levels of FoxP3. This observation is also supported by flow cytometry finding. Inline with our data, Kiniwa et al showed FoxP3 gene expression in both CD4 and CD8 T cells derived from prostate tumor infiltrating lymphocytes (TILs), and also their expression was higher compared to effector T cells [13]. These data clearly suggest that CD8 Treg cells and CD8 T cells derived from TILs express transcription factor FoxP3 but their expression is elevated in cancer patients. In line with flow cytometry and RT-PCR data of FoxP3 expression, we have also shown the expression of FoxP3 by immunoblotting from CD4 Treg cells and CD8 Treg cells.

Functional studies clearly demonstrated that CD8 Treg cells inhibited proliferation of CD4 T cells in a concentration dependent manner. Our observation is consistent with other findings from prostate, colon and nasopharyngeal cancers [13], [14], [15]. Moreover, our data showed that proliferation inhibition of CD4 T cells by CD8 Treg cells isolated from MM patients and healthy donors did not differ significantly. In the absence of CD8 Treg cells, CD4 T cells isolated from MM patients and healthy donors proliferated similarly in proliferation assays. These observations suggest no defects in proliferation potential of CD4 T cells from MM patients but immune suppression in MM patients can be induced in the presence of CD8 Treg cells. Thus, CD8 Treg cells might be a cause for immune impairment in MM patients. Unfortunately, we did not have enough material to compare the ability of suppression by CD4 Treg cells and CD8 Treg cells. However, two studies compared these regulatory cells in prostate and colon cancers, and both studies acknowledged that CD4 Treg cells and CD8 Treg cells were similar in their suppressive function [13], [15].

In line with the finding of inhibition of proliferation, we and Chaput et al found that pro-inflammatory cytokine IFN-γ level was decreased with respect to the number of CD8 Treg cells added to the proliferation assay [15]. We believe that from our and Chaput et al studies, pro-inflammatory cytokine response by T cells could be reduced due to the presence of CD8 Treg cells [15]. To support this conclusion, current study adds evidence from proliferation assays, where the level of IFN-γ was increased in the absence of CD8 Treg cells. In addition, to prove the immunosuppressive activity of CD8 Treg cells, we evaluated IL-10 from culture supernatants. The data showed that depending on the number of CD8 Treg cells added to proliferation assay, level of IL-10 was increased in MM patients and healthy donors. Data from proliferation assays without the presence of CD8 Treg cells showed significant increase in the level of IL-10 from MM patients compared to healthy donors. However, proliferation experiments showed no defect in proliferation potential of CD4 T cells from MM patients in the absence of CD8 Treg cells but we believe that these cells are polarized toward the Th2 cell type, which is evidenced by increased level of IL-10 compared to healthy donors. This observation further supports the idea of immunological impairment in MM patients.

Next, we evaluated the RNA profile of cytokines in CD8 Treg cells stimulated with PMA plus ionomycin from MM patients and healthy donors. Our results showed no significant changes between MM patients and healthy donors; however, IL-2, IFN-γ, TNF-α and TGF-β expression from CD8 Treg cells was increased in healthy donors compared to MM patients. In contrast, IL-10 (immune suppressive cytokine) and IL-6 (enhancer of myeloma cell) expression was increased in MM patients. These data suggest that increased expression of immune suppressive cytokine (IL-10) by CD8 Treg cells isolated from MM patients might enhance the immune suppression. Our data are partially consistent with Li et al finding, who showed that CD8 Treg cells from TILs of nasopharyngeal cancer patients had increased expression of IL-10 and IFN-γ, and low expression of IL-2, TNF-α and TGF-β [14]. In contrast to our and Li et al findings, Cosmi et al showed that activated/stimulated CD8+CD25hi+ thymocytes were incapable of secreting cytokines [11], [14]. Regarding non-regulatory CD8 T cells, MM patients and healthy donors expressed similar RNA profile of cytokines.

When we compared frequency and number of total lymphocytes from MM patients and healthy donors, a significant reduction was observed in MM patients and negative correlation was observed between number of CD8 Treg cells and total lymphocyte counts. Also, similar observation was noticed between CD8 Treg cells and CD4/CD8 ratio but failed to reach statistical significance. These data suggest that impairment in lymphocyte counts of MM patients is associated with elevated number of suppressive CD8 Treg cells, and we believe this might predispose patients to infectious complications. Our previous study and other studies have demonstrated the association of CD4 Treg cells with clinical stages, progression risk and survival outcome of MM [3], [8], [23]. In this study also, we compared CD8 Treg cells with clinical stages and clinical features. The analysis showed insignificant increase in CD8 Treg cells with respect to clinical stages and adverse clinical features (β2- microglobulin, hemoglobin, calcium and plasma cell infiltration) (data not shown).

In conclusion, we showed the presence of a functional regulatory subset expressing CD8 phenotype in MM patients. These CD8 Treg cells expressed antigenic profile similar to CD4 Treg cells, which was evidenced by flow cytometry and RT-PCR data. In MM, CD8 Treg cells were elevated and functionally active in suppressing CD4 T cell proliferation and IFN-γ secretion. Although their clinical relevance is unknown at present, we believe that these CD8 Treg cells enhance immune impairment in MM patients, thereby increasing infectious complications and enhancing disease progression.

## Supporting Information

Table S1 **GAPDH and FoxP3 Ct values of T regulatory and non-regulatory T cells from healthy donors and multiple myeloma patients.**
(DOC)
