# Supplementary material for: Functionally Suppressive CD8 T Regulatory Cells Are Increased in Patients with Multiple Myeloma: A Cause for Immune Impairment
Source: PLoS One. 2012 Nov 13;7(11):e49446. doi: 10.1371/journal.pone.0049446 (PMC3496705; doi:10.1371/journal.pone.0049446)
Supplement: Table S1 — GAPDH and FoxP3 Ct values of T regulatory and non-regulatory T cells from healthy donors and multiple myeloma patients. (DOC) [file pone.0049446.s001.doc]

**Table S1.**

| **Samples** | **CD8 Treg cells** | | **CD4 Treg cells** | | **non-regulatory T cells** | |
| --- | --- | --- | --- | --- | --- | --- |
|  | **GAPDH** | **FoxP3** | **GAPDH** | **FoxP3** | **GAPDH** | **FoxP3** |
| HD1 | 25.220 | 32.158 | 24.421 | 27.860 | 26.730 | 33.405 |
| HD2 | 25.543 | 33.013 | 24.964 | 28.159 | 25.221 | 31.500 |
| HD3 | 24.715 | 30.842 | 25.175 | 28.245 | 25.587 | 33.558 |
| HD4 | 25.274 | 31.192 | 24.949 | 26.472 | 24.998 | 32.744 |
| HD5 | 24.922 | 31.040 | 23.964 | 25.112 |  |  |
| MM1 | 25.641 | 30.316 | 24.437 | 24.307 | 25.724 | 32.011 |
| MM2 | 24.523 | 31.278 | 25.264 | 27.047 | 25.617 | 32.400 |
| MM3 | 25.752 | 30.796 | 25.095 | 27.530 | 25.338 | 34.264 |
| MM4 | 25.062 | 30.778 | 24.060 | 27.853 | 24.683 | 33.236 |
| MM5 | 25.238 | 29.491 | 25.584 | 30.449 |  |  |

Ct values represented above are mean Ct value from duplicates. HD, healthy donor; MM,

multiple myeloma.
